# In-vivo evidence that high mobility group box 1 exerts deleterious effects in the 1-methyl-4-phenyl-1,2,3,6-tetrahydropyridine model and Parkinson's disease which can be attenuated by glycyrrhizin

**DOI:** 10.1016/j.nbd.2016.02.018

**Published:** 2016-07

**Authors:** Matteo Santoro, Walter Maetzler, Petros Stathakos, Heather L. Martin, Markus A. Hobert, Tim W. Rattay, Thomas Gasser, John V. Forrester, Daniela Berg, Kevin J. Tracey, Gernot Riedel, Peter Teismann

**Affiliations:** a School of Medical Sciences, University of Aberdeen, Institute of Medical Sciences, Foresterhill, Aberdeen, AB25 2ZD Scotland, UK; b Center of Neurology, Department of Neurodegeneration, Hertie-Institute for Clinical Brain Research, University of Tuebingen, Hoppe-Seyler-Str. 3, 72076 Tuebingen, Germany; c German Center for Neurodegenerative Diseases (DZNE) Tuebingen, Otfried-Müller-Str. 27, 72076 Tuebingen, Germany; d Ocular Immunology Program, Centre for Ophthalmology and Visual Science, The University of Western Australia, Western Australia 6009, Australia; e Centre for Experimental Immunology, Lions Eye Institute, Nedlands, Western Australia 6009, Australia; f Feinstein Institute for Medical Research, Manhasset, NY 11030, USA

**Keywords:** COX, cyclooxygenase, CSF, Cerebrospinal fluid, DOPAC, 3,4-Dihydroxyphenylacetic acid, GFAP, glial fibrillary acidic protein, H&Y, Hoehn & Yahr, HMGB1, high-mobility group box 1, HVA, homovanillic acid, MPP^+^,  1-methyl-4-phenylpyridinium, MPTP, 1-methyl-4-phenyl-1,2,3,6-tetrahydropyridine, OD, optical density, PD, Parkinson's disease, RAGE, receptor for advanced glycation endproducts, SNpc, substantia nigra pars compacta, TH, tyrosine-hydroxylase, TNF-α, tumour necrosis factor-alpha, Wt, wild type, Parkinson's disease, MPTP, High-mobility group box 1, receptor for advanced glycation endproducts

## Abstract

High-mobility group box 1 (HMGB1) is a nuclear and cytosolic protein that is released during tissue damage from immune and non-immune cells — including microglia and neurons. HMGB1 can contribute to progression of numerous chronic inflammatory and autoimmune diseases which is mediated in part by interaction with the receptor for advanced glycation endproducts (RAGE). There is increasing evidence from *in vitro* studies that HMGB1 may link the two main pathophysiological components of Parkinson's disease (PD), i.e. progressive dopaminergic degeneration and chronic neuroinflammation which underlie the mechanistic basis of PD progression.

Analysis of tissue and biofluid samples from PD patients, showed increased HMGB1 levels in human postmortem substantia nigra specimens as well as in the cerebrospinal fluid and serum of PD patients. In a mouse model of PD induced by sub-acute administration of 1-methyl-4-phenyl-1,2,3,6-tetrahydropyridine (MPTP), systemic administration of neutralizing antibodies to HMGB1 partly inhibited the dopaminergic cell death, and reduced the increase of RAGE and tumour necrosis factor-alpha. The small natural molecule glycyrrhizin, a component from liquorice root which can directly bind to HMGB1, both suppressed MPTP-induced HMGB1 and RAGE upregulation while reducing MPTP-induced dopaminergic cell death in a dose dependent manner.

These results provide first in vivo evidence that HMGB1 serves as a powerful bridge between progressive dopaminergic neurodegeneration and chronic neuroinflammation in a model of PD, suggesting that HMGB1 is a suitable target for neuroprotective trials in PD.

## Highlights

• HMGB1 is up-regulated in Parkinson's disease.
• HMGB1 is translocalized into the cytoplasm after MPTP.
• Inhibition of HMGB1 protects against MPTP-toxicity.
• Translocalization of HMGB1 is reduced after inhibition a neutralizing antibody or glycyrrhizin.

## 1 Introduction

Occurrence of chronic inflammation in Parkinson's disease (PD) is increasingly recognized (Hirsch and Hunot, 2009, Teismann and Schulz, 2004), and recent studies argue in favour of a central role for chronic inflammation in disease progression in different PD models (Gao et al., 2003, Zhang et al., 2010). The basic idea emerging from these observations is as follows: neurons can sustain a certain level of inflammatory challenge; however, if the inflammatory process is prolonged and levels of neurodegeneration exceed a certain threshold; death of neurons, accumulation of protein aggregates, and unregulated neuroinflammation potentiate each other almost independently of the initial cause of neurodegeneration (Gao et al., 2011, Hirsch et al., 2013). In other fields this general phenomenon has been described as dysregulated parainflammation (Medzhitov, 2008, Xu et al., 2009).

High-mobility group box 1 (HMGB1) is a non-histone DNA binding protein, which stabilizes nucleosome formation indirectly, and regulates the interaction of transcription factors with DNA (Bustin and Reeves, 1996). HMGB1 can be secreted actively from inflammatory cells but is released in greater amounts passively from dying cells during tissue damage and inflammatory disease and can then act as a damage associated molecular pattern (DAMP) to initiate inflammatory responses (Andersson and Tracey, 2011). This mechanism may be particularly relevant for PD as a recent study elegantly demonstrated that: by use of cell models reflecting PD pathophysiology HMGB1 signalling indeed triggered progressive dopaminergic neurodegeneration by uncontrolled chronic inflammation (Gao et al., 2011). The underlying mechanisms of action are the increased expression of HMGB1-binding multi-ligand receptors, which are mainly located on the surface of cells of the innate immune system (Andersson et al., 2008) and the activation of these receptors due to translocation of HMGB1 to the cytosol (Lu et al., 2014). HMGB1 binds to receptor for advanced glycation end products (RAGE) (Andersson et al., 2008, Scaffidi et al., 2002), Toll-like receptors 2 (TLR2) and 4 (TLR4) (Andersson et al., 2008, Wahamaa et al., 2011) and macrophage antigen complex 1 (Mac1) (Gao et al., 2011). Of these, the RAGE-HMGB1 interaction has been shown to be pathogenetic, e.g. in models of ischemic injury (Kim et al., 2006) and Alzheimer's disease (Mazarati et al., 2011).

Most recently, a study focusing on the cause of tissue damage during subarachnoid haemorrhage (Sun et al., 2013) suggested that the deleterious effects of released HMGB1 can be modulated by naturally occurring anti-inflammatory compounds such as glycyrrhizin. Glycyrrhizin may be a particularly interesting target compound for neuromodulation in PD, as it is a commonly used sweetener and generally recognized as safe when not excessively consumed (Isbrucker and Burdock, 2006). The European Union suggests that up to 100 mg of glycyrrhizic acid a day, which equals approximately 50 g of liquorice sweets (Stormer et al., 1993) do not represent any health hazard for humans.

Based on these data, and on our recent observation that a second RAGE ligand, S100B, has deleterious effects in the 1-methyl-4-phenyl-1,2,3,6-tetrahydropyridine (MPTP)-model (Sathe et al., 2012), we have explored the role of HMGB1 in the MPTP model as well as in biospecimens from PD patients.

We have found that HMGB1 is up-regulated in the substantia nigra (SN), the cerebrospinal fluid (CSF) and the serum of PD patients, as well as in MPTP-treated mice. In addition, blockade of HMGB1 with a neutralizing antibody in the MPTP model led to reduced levels of expression and activation of both HMGB1 and RAGE associated with reduced gliosis on histological examination, suggesting that, inhibition of HMGB1 may be neuroprotective. Furthermore, we found that the liquorice constituent glycyrrhizin also reduced HMGB1 expression and appeared to be neuroprotective in the MPTP-model.

## 2 Materials and methods

### 2.1 Human brain tissue collection and processing

Human nigral tissue was obtained from the Parkinson UK Brain Bank at Imperial College, London, UK. Selected PD and control samples were matched for age at death. PD patients (n = 6) were 79 ± 2 (mean ± SEM) years with an average disease duration of 9 ± 1 years. Age at onset of disease was 70 ± 2 years. None of the PD patients had a positive family history of the disease. Control cases (n = 5) were aged 80 ± 3 years at death. Post-mortem delay was 38 ± 10 h in PD samples and 25 ± 10 h in control samples (p = 0.38).

Samples were isolated in NP-40 buffer with protease inhibitors (Complete Protease Inhibitor Cocktail Tablets, Roche, UK) 1:20 (wt/vol) and processed by hand homogenisation. Extracts were centrifuged at 14,000 rpm (18,620 ×* g*; Mikro 200R) for 20 min at 4 °C and supernatants retained.

### 2.2 CSF/serum collection, and HMGB1 determination in these body fluids

CSF and serum samples of 75 PD patients and 47 controls collected between 2010 and 2013 were obtained from the Neuro Biobank of the University of Tuebingen. PD patients were diagnosed according to current criteria (Hughes et al., 1992) by movement disorders specialists.

Controls did not have any signs or symptoms for neurodegenerative diseases. Twenty of the controls were investigated by spinal tap performed for investigation of lumbar pain; sixteen of the controls displayed non-specific symptoms such as headache and mood disturbances (exclusive of vascular events), and eleven healthy volunteers underwent spinal taps as healthy controls in the frame of scientific studies. None of the included individuals had a history or clinical signs of inflammation. All patients and controls underwent a thorough neurological examination, and were classified according to the Hoehn & Yahr (H&Y) staging. Patients with dementia were not included. Demographic and clinical data are given in Table 1. All participants gave their informed consent, and the local ethical committee approved the study.

CSF and blood collection as well as determination of routine diagnostic parameters were performed according to a standardized protocol (Maetzler et al., 2011). In brief, spinal taps were performed between 8:00 and 10:00 in the morning under fasting conditions. Samples were centrifuged and stored at − 70 °C within 1 h of sample collection. Only samples from donors with normal CSF values (< 4 cells/μl, CSF albumin levels < 500 mg/l) were included. Routine CSF and serum parameters (albumin, immunoglobulin G, cell count) did not differ significantly between PD and controls (Table 1). HMGB1 was measured in duplicate with a commercially available ELISA kit (ST51011, IBL, under licence of Shino-Test Corporation), according to the manufacturer's instructions. The minimum detection level was 0.3 ng/ml.

### 2.3 Animals, induction of PD-like disease and interventions

Wild type (WT) C57BL6J mice (obtained from Charles River Laboratories; 3–6 mice per time point) were injected intraperitoneally (i.p.) with MPTP (30 mg/kg i.p., Sigma, UK) dissolved in saline, once a day for over 5 consecutive days. Some animals also received HMGB1-neutralizing antibody (200 μg) (Yang et al., 2004) i.p. prior to treatment with MPTP and every third day afterwards. Control mice received saline only. Glycyrrhizin (16.8 or. 50 mg/kg, Sigma) was administered i.p. 30 min after the first injection of MPTP once daily at 24 h intervals over the course of the study, the dose was based on previous studies (Abe et al., 2008, Kim and Lee, 2008).

Animals were sacrificed at selected time points after the last injection of MPTP (0, 1, 2, 4, 7, 14, 21 days). For HPLC measurements and tyrosine hydroxylase (TH) immunohistochemistry, mice were sacrificed 21 days after the last MPTP injection. All protocols were in accordance with the Home Office regulations.

### 2.4 Western blot analysis

Mouse and human brain extracts were prepared and Western blot analyses performed as described earlier (Sathe et al., 2012). Primary antibodies were: HMGB1 (1:1000, Millipore UK); RAGE (1:1000, Millipore); S100B (1:1000, Sigma); cyclooxygenase-2 (COX-2) (1:250, BD Bioscience, UK); tumour necrosis factor-alpha (TNF-α) (1:1000, Abcam, UK); β-actin (1:25,000, Sigma). Blots were incubated at 4 °C overnight. Horseradish peroxidase-conjugated secondary antibodies (anti-rabbit or anti-mouse 1:10,000, Amersham) and ECL solution (Luminol sodium salt in 0.1 mM Tris HCl and Para-hydroxycoumarin in DMSO) were used for chemiluminescence detection. Bands were quantified using the FluorChem 8800 digital image system (Alpha Innotech, UK).

### 2.5 Total RNA extraction and RT-PCR

Total RNA from the ventral midbrain was isolated in Trizol (Invitrogen, UK). First strand cDNA was analysed using the Superscript II kit (Invitrogen) following manufacturer's instructions. The cDNA was then amplified by polymerase chain reaction in a 10 μl total reaction volume using the Roche Light cycler 480. The primer mouse sequence used was as follows: HMGB1 5′-TCGCCTTTGATTTTGGGGCGGT-3′ (forward) and 5′-AGCTGAGAATGGCTTGGGTCGT-3′ (reverse). As internal control, β-actin cDNA was co-amplified using primer sequences 5-TGTGATGGTGGAATGGGTCAG-3′ (forward) and 5′-TTTGATGTCACGCACGATTTCC-3′ (reverse). All primers were mouse-specific and intron-spanning and were designed based on reported sequences available from the GENEBANK database. The PCR products were all of the expected size, and their proper identities were confirmed by automatic sequencing performed by DNA Sequencing & Services (MRCPPU, College of Life Sciences, University of Dundee, Scotland, www.dnaseq.co.uk) using Applied Biosystems Big-Dye Ver 3.1 chemistry on an Applied Biosystems model 3730 automated capillary DNA sequencer.

### 2.6 Immunohistochemistry

Immunostaining was performed according to standardized in-lab protocols (Sathe et al., 2012). Primary antibodies (in PBS-Triton-NGS) were: HMGB1 (1:1000, Sigma); glial fibrillary acidic protein (GFAP) (1:1000, DAKO); TH (1:1000, Millipore) and Iba1 (1:500, Wako Chemicals, Germany). The sections were incubated in anti-rabbit (1:200, cy3, Jackson Immuno Research, UK) or anti-mouse (1:300, Alexa Fluor 488 nm, Molecular Probes, UK) fluorescein-conjugated antibodies for visualization using confocal microscopy. Immunohistochemistry was carried out as described earlier (Sathe et al., 2012) and counterstained for Nissl (Thionin, Sigma). TH-, GFAP-, Iba1- and Nissl-positive cells in the SN pars compacta (SNpc) were counted using the optical fractionator method with the examiner being blinded towards treatment group. Striatal density of TH immunoreactivity was measured as described elsewhere (Sathe et al., 2012). For double immune-fluorescence microglia were incubated with first biotinylated *Lycopersicon esculentum* (tomato) lectin. (Vector Laboratories, Peterborough, UK) and revealed with streptavidin conjugated with DyLight 488 (Vector Laboratories).

### 2.7 HPLC

Levels of 1-methyl-4-phenylpyridinium (MPP^+^) dopamine and its metabolites, 3,4-dihydroxyphenylacetic acid (DOPAC) and homovanillic acid (HVA) were measured in striatal samples as described (Sathe et al., 2012).

### 2.8 Data analysis

Data were analysed with Graphpad Prism (version 5.04) and JMP software (version 10.0, SAS). All values are expressed as means ± the standard error of the mean (SEM) or frequency unless stated otherwise. Differences between means were analysed using Student's t-test (2 groups) and the one-way ANOVA (> 2 groups). When ANOVA showed significant differences, pair-wise comparisons between means were assessed using the Newman–Keuls post-hoc test. Pearson correlation coefficient was used for correlation analyses, and the Fisher's exact test for comparison of categorical data. Differences of HMGB1 levels between PD patients and controls were calculated using a linear regression model (two effects: PD yes/no, and age), with the likelihood ratio as outcome. Differences were assumed to be significant at p < 0.05 (two-sided).

## 3 Results

### 3.1 HMGB1 is detectable in neuronal and glial cells of human post-mortem tissue, and higher levels are present in PD patients than controls

**Supplementary Fig. 1.**
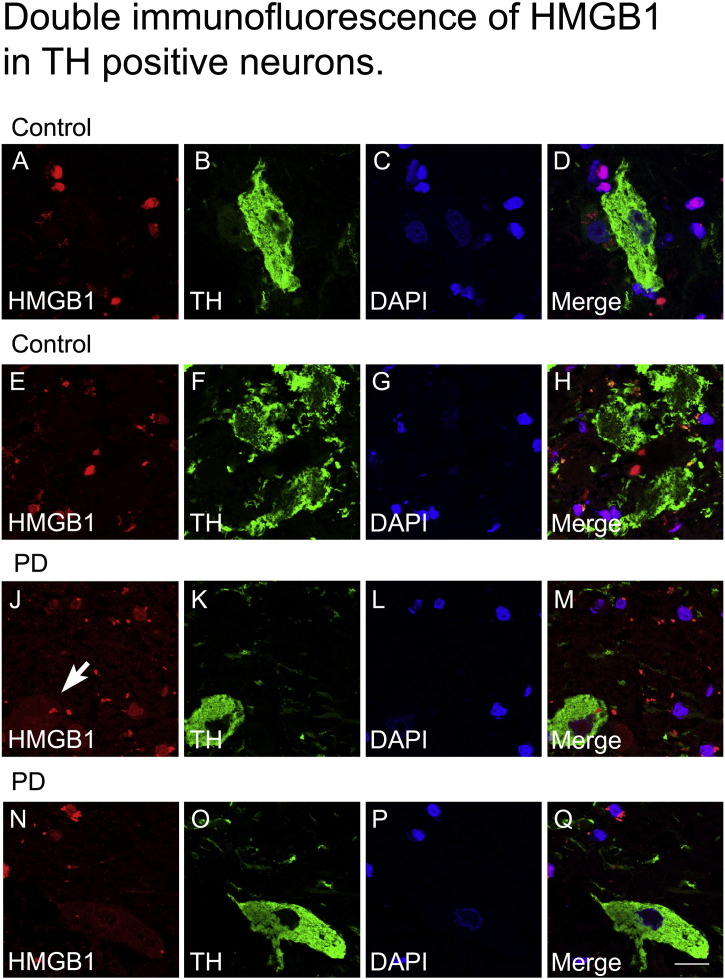
Double immunofluorescent staining for TH positive neurons (green) and HMGB1 (red) in human post mortem SNpc of control and PD cases. Occurrence of protein translocation and localization of HMGB1 within the cytoplasmic compartment of TH positive neurons (arrow) is observed in PD samples (J-Q) but not in control samples (A-H). Nuclei are stained with DAPI (blue), scale bar = 20 µm.

**Supplementary Fig. 2.**
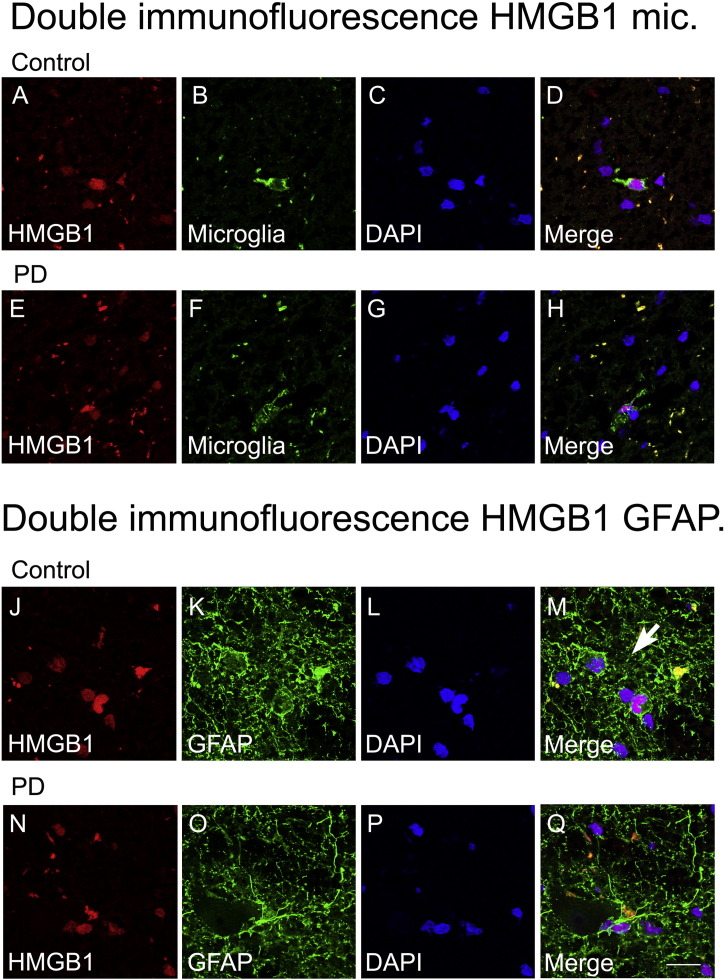
Double immunofluorescence for microglia (green) and HMGB1 (red) (A-H), and GFAP (green) and HMGB1 (red) (J-Q) in human post mortem SNpc of control cases and PD cases. HMGB1 nuclear translocation is not observed in microglia (E-H) and GFAP positive astrocytes (N-Q) of PD samples. Nuclei are stained with DAPI (blue), scale bar = 20 µm.

**Fig. 1:**
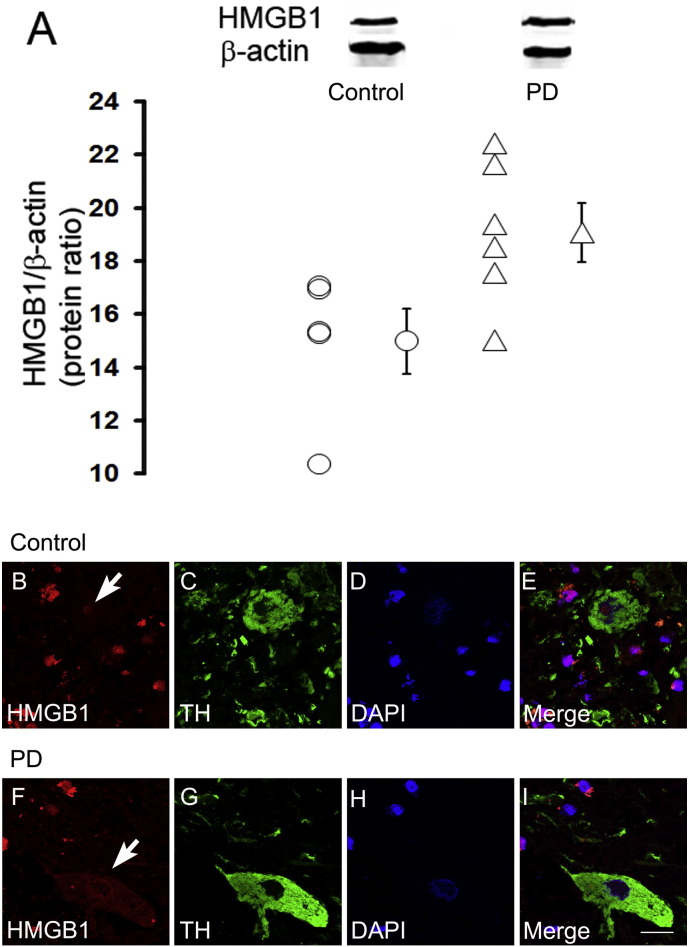
HMGB1 in dopaminergic neurons of human post-mortem substantia nigra (SNpc). Using western blotting, HMGB1 protein levels in SNpc are, as a mean, higher in six samples of Parkinson's disease (PD), compared with five control samples (A). Double immunofluorescence studies reveal cytoplasmic HMGB1 localization in TH-positive dopaminergic neurons of patients with PD (F–I), but not in control patients (B–E). *p < 0.05. Scale bar = 20 μm.

Immunostaining in the post-mortem midbrain slices of PD and control subjects revealed that HMGB1 protein is consistently expressed in TH-positive neurons within the SNpc (Fig. 1B–I). PD patients regularly showed cytosolic location of HMGB1 but not controls, indicating translocation of HMGB1 into the cytosol in TH-positive neurons (Fig. 1F–I, white arrow). Immunoblotting yielded significantly higher HMGB1 protein levels in the SNpc of PD cases than in those of controls (Fig. 1A). For additional information double immunofluorescence labelling of HMGB1 along with three different cell markers, TH (Supplemental Fig. 1), GFAP and Microglial cells (using tomato lectin) (Supplemental Fig. 2) in human brain tissue was performed.

### 3.2 Heightened serum and CSF HMGB1 levels in PD

**Fig. 2:**
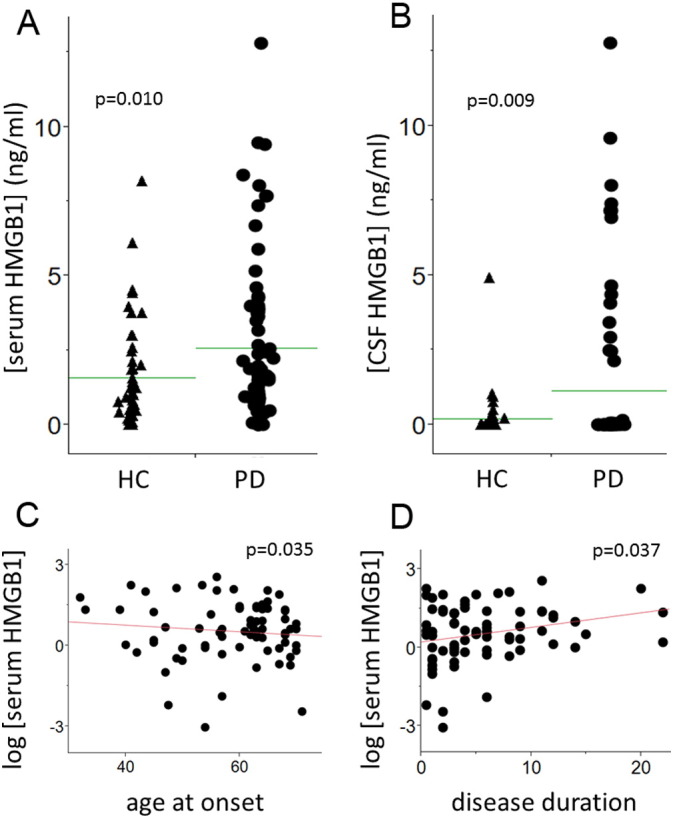
HMGB1 levels in serum and cerebrospinal fluid in patients with Parkinson's disease. In the immunoassay, HMGB1 levels are significantly increased in both serum (A) and CSF (B) of 75 patients with Parkinson's disease (PD) compared to 47 controls (HC). HMGB1 serum levels of PD patients are negatively correlated with age at onset (C), and positively with disease duration (D).

In both serum and CSF of the 122 sample pairs investigated, HMGB1 levels were significantly higher in PD patients than in controls (Fig. 2A, B). In PD patients, serum (p = 0.035, Fig. 2C), but not CSF HMGB1 levels (p = 0.75) correlated negatively with age at onset of the disease. Serum HMGB1 levels showed a significant positive correlation with disease duration (p = 0.037, Fig. 2D) which was not the case in CSF samples (p = 0.80).

No relevant associations were detected between histopathological staging of disease (H&Y stage) and serum (p = 0.52) or CSF HMGB1 levels (p = 0.89), respectively. Table 1 presents details of the demographic, clinical and biochemical parameters of the cohorts.

### 3.3 HMGB1 RNA and protein levels are increased after MPTP treatment

**Fig. 3:**
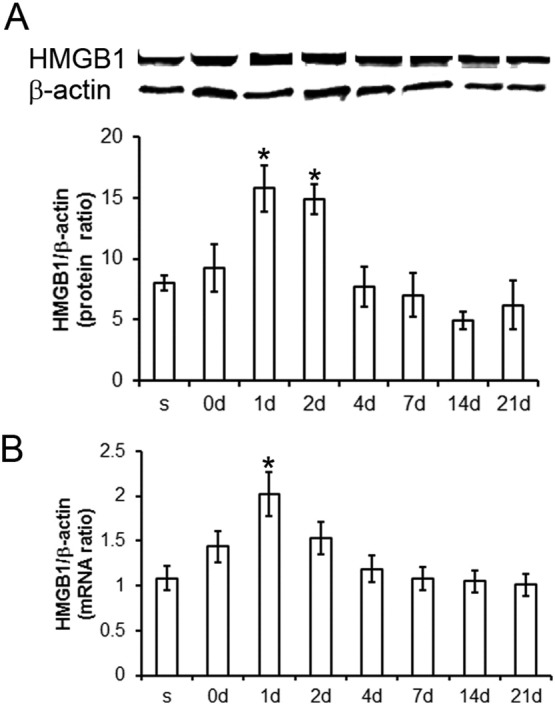
HMGB1 mRNA and protein levels are upregulated after MPTP treatment. In mice, real-time PCR and Western blot tests indicate that HMGB1 mRNA and protein levels are increased at day 1 after MPTP treatment. Within 4 days, HMGB1 protein (A) and mRNA levels (B) return back to initial values. Data are mean ± SEM, n = 4–6 mice per group. *p < 0.05; ANOVA with student Newman–Keuls post-hoc test.

**Fig. 4:**
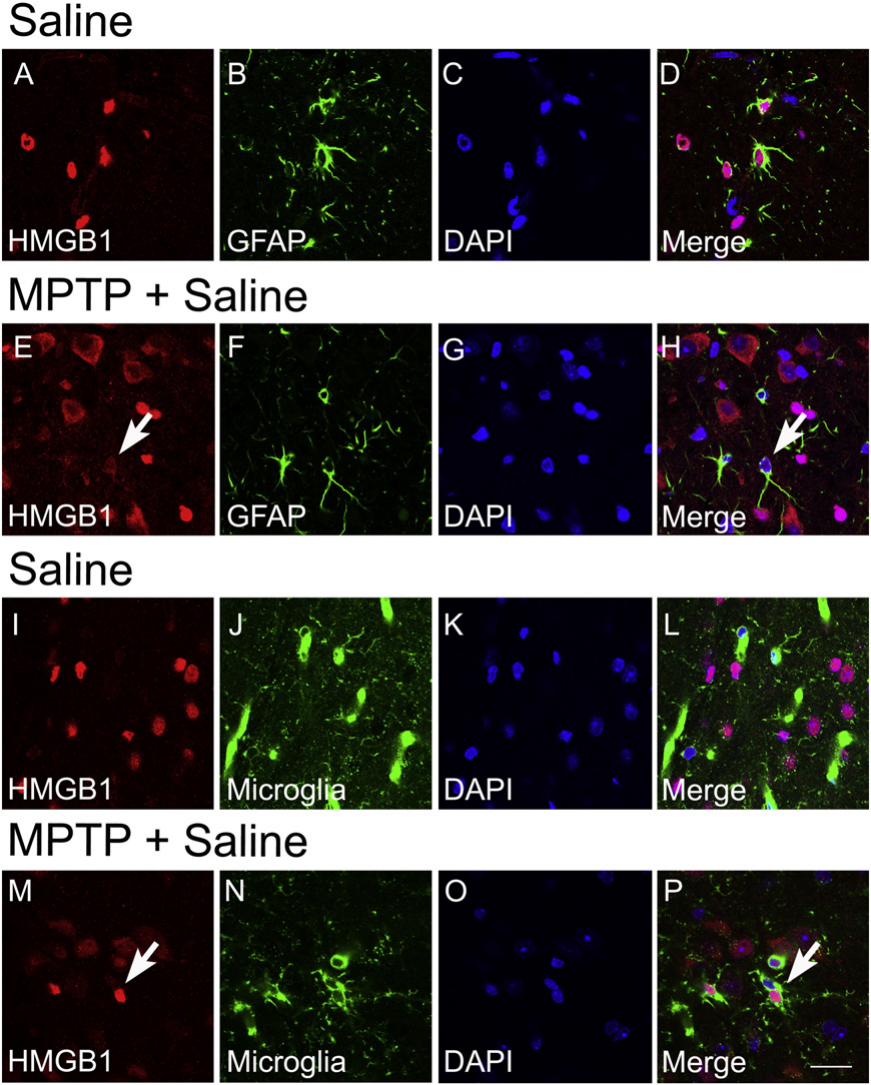
Double immunofluorescence reveals localization of HMGB1 in the nuclei with translocation to the cytosol after MPTP in GFAP-positive astrocytes (E–H) and Iba-1-positive microglia (M–P) in the substantia nigra pars compacta (2d after MPTP). Data are mean ± SEM, n = 4–6 mice per group. Scale bar = 20 μm.

**Fig. 5:**
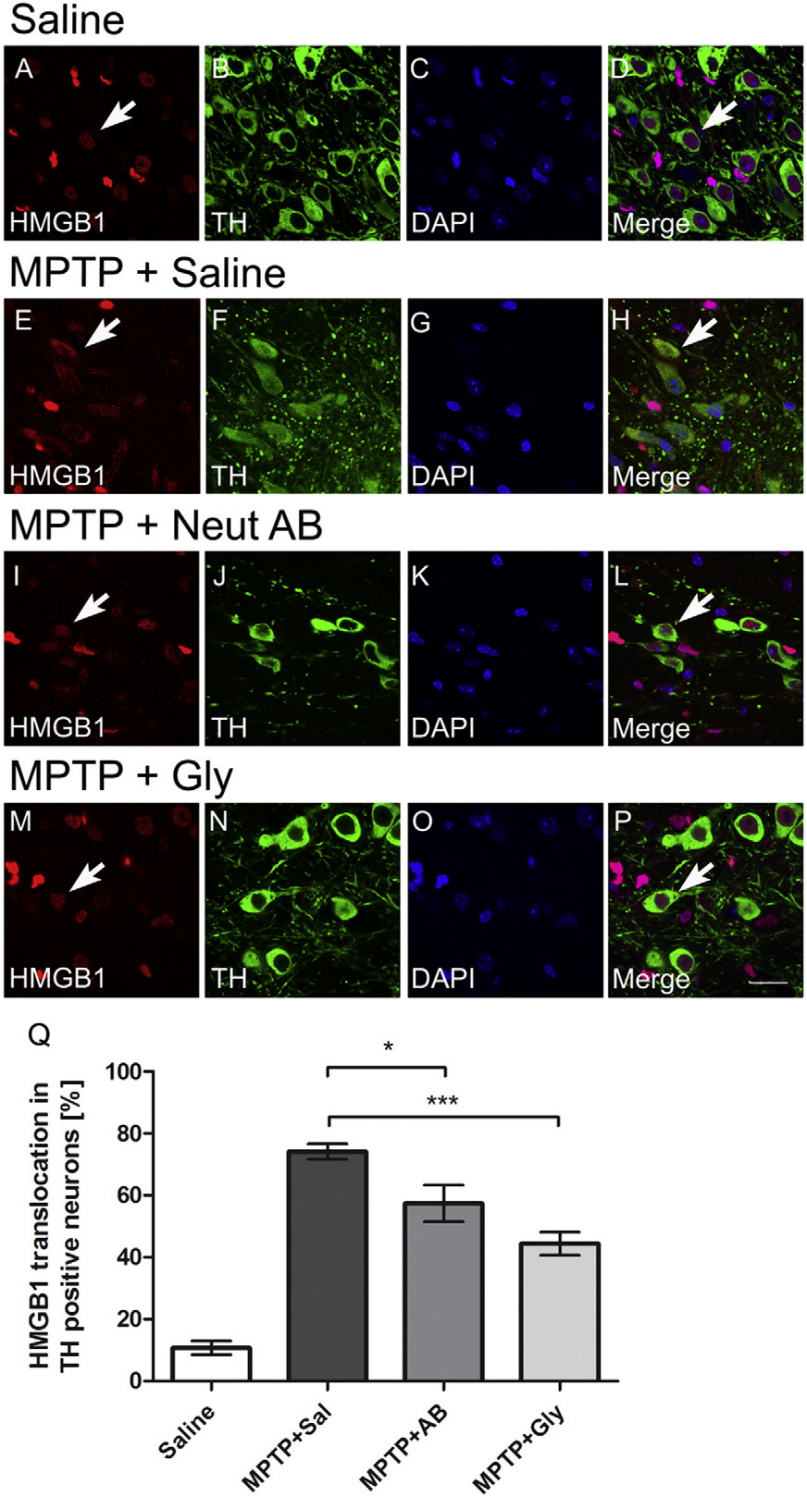
HMGB1 is translocated into the cytoplasm 1 day after MPTP treatment in dopaminergic neurons. Double immunofluorescence in mouse midbrain reveals localization of HMGB1 in the nuclei of TH-positive dopaminergic neurons after saline (A–D, white arrow), with a clear translocation to the cytosol after MPTP (E–H, white arrows), which is significantly reduced in mice receiving HMGB1-neutralizing antibody (I–L) or glycyrrhizin (M–P) (2d after MPTP). (Q) Stereological cell counting in epifluorescence field was performed for TH-positive neurons in SNpc. TH-positive cells presenting staining for HMGB1 in the cytoplasm portion were considered positive to HMGB1 translocation from nuclei to cytoplasm. *p < 0.05, ***p < 0.001 compare to MPTP mice group treated with saline (Newman–Keuls post-hoc test). Data are generated with 5 mice per group. Value are presented with mean ± SEM. Scale bar = 20 μm.

In mice, mRNA and protein levels of HMGB1 in the ventral midbrain (the brain region containing the SNpc) are increased one day after MPTP administration and then returned back to baseline levels (Fig. 3A, B). Immunohistochemistry confirmed HMGB1 as a protein widely expressed in astrocytes and microglia (Fig. 4). Dopaminergic neurons clearly showed enhanced staining for HMGB1, due to increased levels in the cytosol, indicating that HMGB1 is more actively translocated from the nucleus to the cytoplasm one day after in MPTP-treated mice than in saline-treated mice (Fig. 5A–H, white arrows), which is significantly reduced in mice receiving HMGB1-neutralizing antibody (I–L, Q) or glycyrrhizin (M–P, Q).

### 3.4 Neutralizing HMGB1 antibody reduces MPTP neurotoxicity

**Fig. 6:**
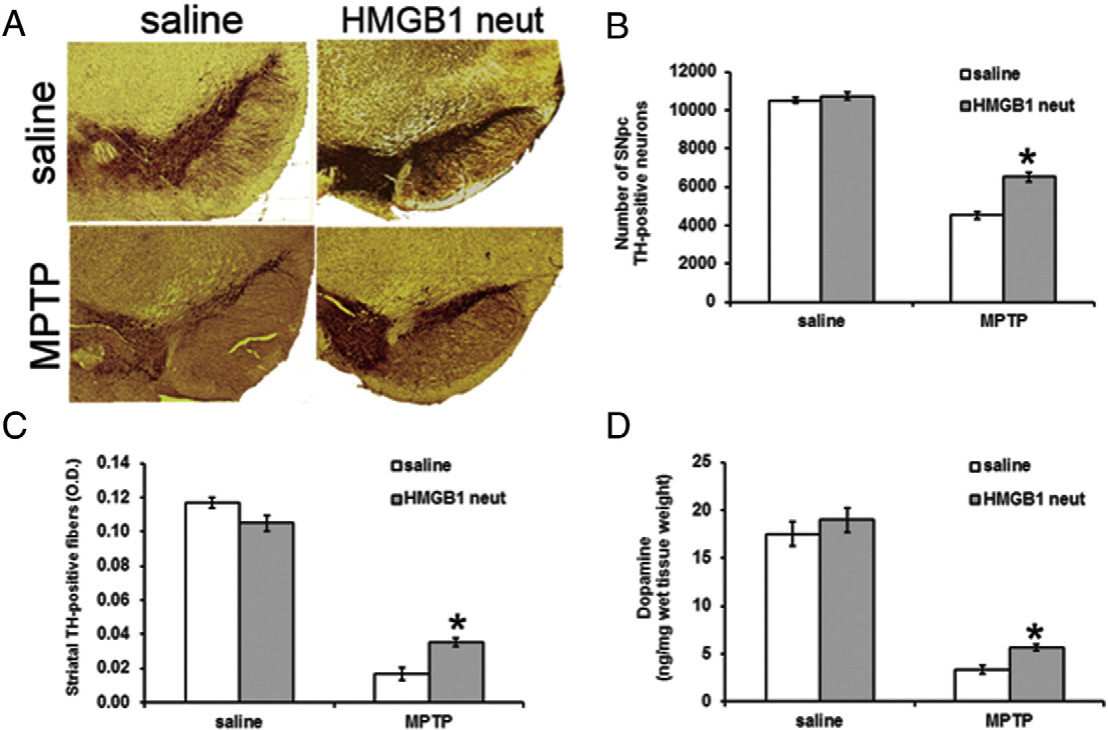
Neutralizing HMGB1 rescues dopaminergic neurons from MPTP toxicity. Numbers of TH-positive neurons in the SNpc are comparable between saline-treated mice receiving and not receiving HMGB1-neutralizing antibody (upper panels; A–B). Three weeks after MPTP treatment, mice initially treated with HMGB1-neutralizing antibody show higher numbers of TH-positive neurons, than do mice not receiving the antibody (lower panel in A and B). Accordingly, depletion of striatal dopaminergic fibres and striatal dopamine depletion assessed three weeks after MPTP are significantly less severe in mice receiving HMGB1-neutralizing antibody (C, D). Cell counts were performed with stereology. OD was assessed by use of ScionImage Scion Corp., Frederick, Maryland, USA). Striatal monoamine levels were assessed by HPLC. *p < 0.05, compared to respective, MPTP treated, mice groups which did not receive HMGB1 antibody (Newman–Keuls post-hoc test). Data are generated with six mice per group, values are presented with mean ± SEM. AB, antibody.

Next we assessed the effects of blocking HMGB1 on the level of disease in the MPTP mouse model. Mice were administered anti-HMGB1 antibody as described in Methods. Significantly more TH-positive neurons (58% in mice receiving neutralizing HMGB1 antibody versus 44% in control mice) as well as Nissl-stained neurons (79% in mice receiving neutralizing HMGB1 antibody versus 59% in control mice) survived after MPTP treatment compared to untreated (Fig. 6A, B and supplemental table 1). This difference was reflected by a significantly less marked decrease in both the density of TH-positive fibres (44 versus 14%, Fig. 6C), and in dopamine levels (30% versus 18%, Fig. 6D) in the striatum of mice treated with neutralizing HMGB1 antibody (Fig. 6C and supplemental table 1). Determination of 1-methyl-4-phenylpyridinium (MPP^+^) levels in the striatum after MPTP treatment with and without concomitant HMGB1 antibody administration indicated that the antibody injection did not impair MPTP metabolism (Table 2).

### 3.5 Neutralization of HMGB1 lowers RAGE and TNF-α levels in MPTP-treated mice

**Fig. 7:**
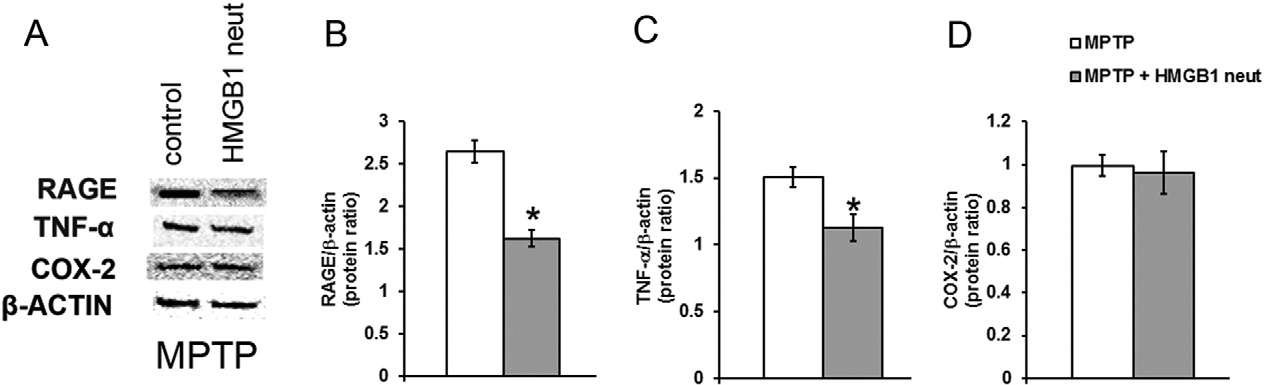
HMGB1 influences MPTP toxicity via RAGE and TNF-α. Two days after MPTP treatment, immunoblotting reveals higher levels of RAGE (A,B) and TNF-α protein (A,C) in mice initially also receiving HMGB1-neutralizing antibody, compared to mice not receiving the antibody. COX-2 protein levels are comparable between the differentially treated groups (A, D). Data are mean ± SEM, n = 4–6 mice per group. *p < 0.05; ANOVA with student Newman–Keuls post-hoc test.

Assessment of the influence of HMGB1 neutralizing antibody on MPTP-induced effects in the mouse midbrain by use of immunoblotting (Fig. 7A) revealed significantly lower RAGE protein levels in mice treated with HMGB1 antibody, compared to mice only receiving MPTP (Fig. 7B). Comparable results were obtained for TNF-α (Fig. 7C). In contrast, COX-2 protein levels were not significantly different between the groups after MPTP administration (Fig. 7D). There were no significant differences in RAGE and TNF-α levels between saline treated groups (data not shown).

### 3.6 Neuronal damage and microglial cell activation in MPTP-treated mice is reduced when HMGB1 is neutralized

As HMGB1 is linked to inflammation, we assessed whether the protective effect of a neutralizing HMGB1 antibody is associated with a reduction of MPTP-induced microglial and astroglial cell activation. Stereological counting showed that, compared to untreated mice, treatment of MPTP-mice with neutralizing antibody to HMGB1 prevented both microglial cell activation (Iba1 positive cells) and gliosis (GFAP positive cells) in the SNpc (Table 3).

### 3.7 Glycyrrhizin reduces MPTP toxicity dose-dependently

**Fig. 8:**
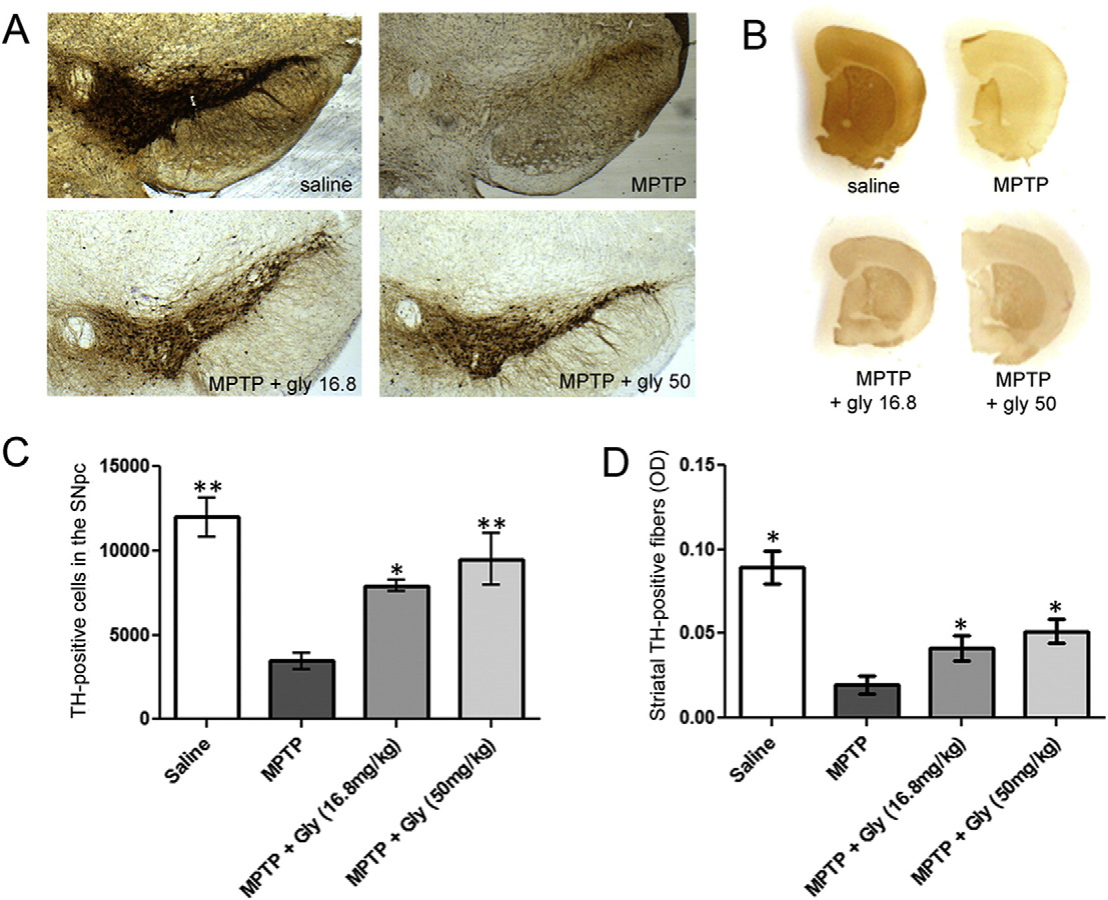
Glycyrrhizin rescues dopaminergic neurons dose-dependently from MPTP toxicity. Mice receiving initial i.p. glycyrrhizin show higher numbers of TH-positive neurons in the SNpc (A, C) as well as more striatal dopamine-positive fibres (B, D), than do mice without glycyrrhizin treatment three weeks after MPTP treatment. This effect is dose-dependent (A-D). Cell counts were performed with stereology. Optical density of striatal TH positive fibres was assessed by use of Scion Image Scion Corp., Frederick, Maryland, USA). Striatal monoamine levels were assessed by HPLC. *p < 0.05, **P < 0.01 compared to respective mice groups which did not receive glycyrrhizin. Data are generated with six mice per group. Values are presented with mean ± SEM. n = 4–5 mice per group; ANOVA with student Newman–Keuls post-hoc test.

We next assessed whether the natural liquorice extract glycyrrhizin, which is known to bind to HMGB1 had any neuroprotective effect in the MPTP mouse model. Glycyrrhizin had a dose-dependent effect on MPTP-induced toxicity in mice after three weeks of treatment (Fig. 8). In mice treated with MPTP alone, 32% of TH-positive neurons (Fig. 8A, C) in the SNpc, and 74% of the Nissl-positive neurons survived. In MPTP-treated mice who received daily doses of 16.8 mg/kg glycyrrhizin, 66% of TH-, and 84% of Nissl-stained neurons survived. Daily i.p. injection of 50 mg/kg glycyrrhizin led to a survival rate of 75% of the TH-positive neurons (Fig. 8A, C), and 84% of the Nissl-stained neurons. These results were confirmed by optical density measures of TH-positive fibres in the striatum. In untreated mice, MPTP caused 80% loss of TH-positive striatal fibres, but in glycyrrhizin-treated mice this was reduced to 53% and 37% loss of fibres at doses of 16.8 mg/kg and 50 mg/kg respectively. (Fig. 8B, D).

To assess whether glycyrrhizin also influences striatal dopaminergic dysfunction after MPTP treatment, dopamine, DOPAC and HVA levels were determined in mouse striatal tissue (see supplemental table 2). The data support a role for HMGB1 in causing damage to the dopaminergic system in MPTP treated mice, since application of glycyrrhizin with increasing doses led to a dose dependent recovery of metabolites levels aforementioned. Control experiments revealed that glycyrrhizin per se did not influence the dopaminergic system. Detailed information about MPP^+^ levels are provided in Table 2.

**Fig. 9:**
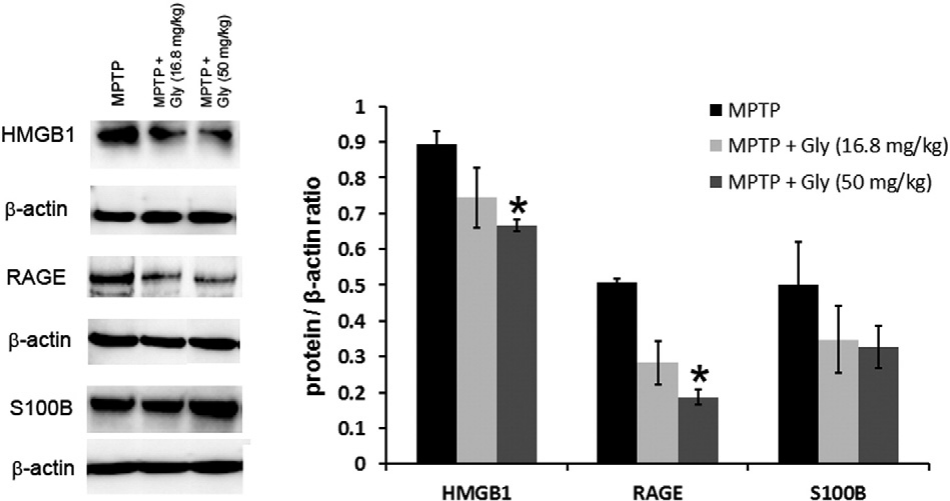
Glycyrrhizin significantly reduces MPTP-induced HMGB1 and RAGE but not S100B levels. In the immunoblot of the ventral midbrain area containing the SNpc, levels of HMGB1 and RAGE proteins one day after MPTP treatment are reduced in mice also receiving i.p. glycyrrhizin. This effect was dose-dependent and reached significance in the group receiving the high dose of glycyrrhizin. S100B, another ligand of RAGE, did not show these changes. Low levels of RAGE, TNF-α and COX-2 were detected in control mice receiving saline (data not shown). Data are mean ± SEM, n = 4–6 mice per group. *p < 0.05; ANOVA with student Newman–Keuls post-hoc test.

The effect of glycyrrhizin also appeared to be mediated via blockade of HMGB1 protein is supported by the evidences of reduced HMGB1 and RAGE protein levels in the midbrain of MPTP mice treated with glycyrrhizin (Fig. 9). Interestingly, levels of a RAGE co-ligand, S100B (Sathe et al., 2012), were not significantly altered.

## 4 Discussion

This is, to the best of our knowledge, the first study (i) showing in vivo evidence that HMGB1 protein levels are altered in patients with PD and that blocking HMGB1 in an animal model of PD is neuroprotective, (ii) providing evidence of how HMGB1 contributes to the progression of the disease by causing activation of microglia and increased gliosis in the SNpc, and (iii) demonstrating that the damaging effects of HMGB1 can be reduced by administration of the naturally occurring HMGB1-binding compound glycyrrhizin.

Increased levels of HMGB1 protein in the SN, as well as in the CSF and serum of PD patients support the hypothesis that HMGB1 is involved in the pathogenesis of PD. The negative correlation of HMGB1 serum levels with age at onset of the disease suggests a specific effect of the HMGB1 in PD pathogenesis; as, even though indirectly, earlier occurrence of the disease is associated with a more “specific” pathology leading to clinical symptoms (de la Fuente-Fernández et al., 2011). However, age at onset parameter shows only 1% (R^2^ = 0.01) of the variance of the HMGB1 serum levels, so the negative correlation is weak. The positive correlation of HMGB1 serum levels (R^2^ = 0.06) with PD duration, shows a variance of 6% of HMGB1 levels. This provide evidences in favour of an involvement of the protein in the previously proposed uncontrolled inflammatory process in PD which, in concert with dying neurons and pathological protein aggregates, leads to a vicious cycle of continuous neurodegeneration (Gao et al., 2011, Hirsch et al., 2013). Moreover, this positive association of HMGB1 levels with disease duration argues for the existence of additional sources beyond damaged and dying neurons in particular at advanced disease stage where the number of potentially HMGB1-delivering (dopaminergic) neurons is reduced as the disease progresses. HMGB1 was consistently observed in the cytosol of TH-positive neurons in PD tissue, but not in control tissue. Additionally MPTP-treated but not saline-treated mice also regularly showed HMGB1 located in the neuronal cytoplasm, indicating that translocation of HMGB1 from the nucleus to the cytoplasm is increased in these animals. Although the process of HMGB1 secretion is not entirely clarified yet, we consider this process paramount for the interpretation of our findings (Lu et al., 2014). HMGB1 in the ageing brain has been considered as an inflammatory mediator, and high HMGB1 levels are associated with diverse mechanisms eventually leading to progressive neuronal damage (Kim et al., 2006, Fang et al., 2012, Muhammad et al., 2008, Takata et al., 2012).

The deleterious effects of HMGB1 induced by MPTP can be inhibited by blocking HMGB1, supporting a role for HMGB1 in causing damage to the dopaminergic system after MPTP treatment. In a recent study, Sasaki and colleagues showed that HMGB1 inhibition via monoclonal antibody administration protects against 6-OHDA neurotoxicity in a rat model. Moreover in support of our findings they showed reduced translocation of HMGB1 from nuclei to cytoplasm after treatment with neutralizing antibody (Sasaki et al., 2016). The observed effects in this study were, at least partly, driven by upregulation and activation of the multi-ligand receptor RAGE. We found that neutralization of HMGB1 in the chronic MPTP mouse model led to reduced levels of RAGE and TNF-α in combination with reduced dopaminergic damage, which indicates that the downstream signalling cascades of RAGE, involving NF-kB, contribute to the damaging effect of HMGB1 (Ramasamy et al., 2005). Also, prior studies have implicated HMGB1 exposure in up-regulating RAGE (Andersson and Tracey, 2011, Schmidt et al., 2001), thus inhibition of HMGB1 was likely the cause for the observed reduced expression of RAGE.

Further downstream cascades known to be activated upon ligand–RAGE interaction – such as erk1/2 (p44/p42) MAP kinases, p38 and SAPK/JNK MAP kinases and the JAK/STAT pathway – as well as additional multi-ligand receptors (e.g. TLR 2 and 4, Mac1 receptor (Gao et al., 2011, Andersson et al., 2008, Wahamaa et al., 2011)) are likely to contribute to the HMGB1-mediated effect (Ramasamy et al., 2005). Indeed, Mac1 expressed on the surface of microglia is a promising candidate for additional HMGB1-mediated dopaminergic toxicity, as HMGB1 has recently been shown to induce the production of multiple inflammatory and neurotoxic factors via microglial Mac1 (Gao et al., 2011), and neutralization of HMGB1 as performed in this study dramatically reduced MPTP-induced gliosis. The cytosolic localization of HMGB1 in microglia also suggests that not only HMGB1 released from dying dopaminergic neurons but HMGB1 released from microglia contribute to the observed HMGB1-induced cell death.

HMGB1 mediated activation of RAGE leads to microglia recruitment in a model of stroke (Muhammad et al., 2008). Additionally, a recent work on HMGB1 implicates its oxidation state in inflammatory pathogenesis, such that the oxidative damage associated with PD onset may lead to conversion of the nuclear thiol HMGB1 to oxidized HMGB1, which would enhance its inflammatory potential (Andersson and Tracey, 2011).

Moreover, mechanisms beyond receptor activation may contribute to the deleterious effects of HMGB1. HMGB1 is detectable in beta amyloid (Aβ) plaques, and high levels of HMGB1 are associated with impaired microglial phagocytosis of amyloid-beta1–40 (Takata et al., 2012) and 1–42 (Takata et al., 2004) in the rat brain. Impaired microglial phagocytosis as shown in the above models leads to delayed clearance of the Aβ species and accelerated neurodegeneration. This is of interest as (i) HMGB1 has been demonstrated to bind to native (Song et al., 2014) and aggregated α-synuclein (Lindersson et al., 2004), and to be present in α-synuclein filament-containing Lewy bodies (Lindersson et al., 2004), and (ii) α-synuclein can be released from neurons, and this extracellular α-synuclein may even more effectively contribute to progressive neurodegeneration, spreading of α-synuclein pathology, and increased neuroinflammation, than intracellular α-synuclein does (Lee et al., 2014). Although the interaction of (secreted) HMGB1 and (secreted) α-synuclein is still hypothetical, we feel that, with the influence of HMGB1 on neuroinflammation and neurodegeneration in the MPTP model shown here, further in-depth studies on the interaction of HMGB1 with different α-synuclein species are urgently needed.

## 5 Conclusion

In agreement with the results of previous studies showing a protective effect of glycyrrhizin in HMGB1-mediated injury (Musumeci et al., 2014), our findings show a substantial reduction of MPTP-induced dopaminergic neurodegeneration, due to inhibition of HMGB1 expression and translocation, accompanied by reduced RAGE levels in midbrain mouse tissue. The dose-dependency of the protective effect of glycyrrhizin observed here indicates that the compound is a finely tuneable inhibitor. This makes it, in our view, a promising candidate for future controlled trials on neuromodulation in PD.

This study shows that HMGB1 is involved in PD, and pharmacological inhibition of HMGB1 by use of neutralizing HMGB1 antibody as well as glycyrrhizin in the MPTP model is neuroprotective.

The following are the supplementary data related to this article.

## Competing interests

Dr. Tracey has a patent Inhibitors of HMGB1 issued to Merck Serono. All other authors declare that they have no competing financial interests.

## Author contributions

WM, PT conceived and designed the experiments, performed experiments, were involved in drafting and editing the manuscript, and interpreted primary data. MS, PS, HLM, MAH, TWR, performed the experiments. WM, TG, GR, JVF, DB, PT edited the manuscript. KJT contributed reagents. All authors read and approved the final manuscript.

## Figures and Tables

**Table 1:** Demographic, clinical and biochemical data of the 122 participants donating CSF and serum for HMGB1 protein analysis.

	PD	Controls	P value
Individuals (f)	75 (53)	47 (27)	1.00
Age at examination [years]	64 ± 9	61 ± 11	0.04
Aao parkinsonism [years]	59 ± 10		
Parkinson duration [years]	5.5 ± 5.0		
Hoehn & Yahr stage (0–5)	2 ± 1		
CSF albumin [pg/mL]	249 ± 84	236 ± 81	0.43
CSF Abeta_1–42_ [pg/mL]	784 ± 289	762 ± 325	0.74
CSF total tau [pg/mL]	198 ± 117	221 ± 136	0.38
CSF phospho-tau [pg/mL]	40 ± 16	42 ± 18	0.44
CSF HMGB1 [ng/mL]	1.14 ± 2.65	0.20 ± 0.74	0.009
Serum HMGB1 [ng/mL]	2.58 ± 2.69	1.59 ± 1.73	0.010

As HMGB1 levels were not normally distributed, they were log-transformed for statistical analysis. Continuous data are presented with mean ± SEM, dichotomous data with frequency. Significance was calculated with Student's t test and Fishers exact test. HMGB1 comparisons were performed using a linear regression model with age as a covariate. Aao: age at onset.

**Table 2:** 1-Methyl-4-phenylpyridinium (MPP^+^) levels.

	MPP^+^ [μg/g tissue]
MPTP + saline treatment	12.31 ± 2.9
MPTP + HMGB1 antibody treatment	10.49 ± 1.9
MPTP + glycyrrhizin	11.30 ± 1.4

MPP^+^ levels in striatum 90 min after MPTP treatment. Values are means ± SEM for four mice per group. Groups were compared with Newman–Keuls post-hoc test (n.s.).

**Table 3:** Effect of HMGB1 neutralization on MPTP-induced astrocytic and microglial markers.

	PD model	Neuroprotection	
No Anti-HMGB1 AB	Anti-HMGB1 AB
Iba1-positive microglial cells	Saline	5554 ± 52	5435 ± 105
MPTP	8084 ± 41⁎	5797 ± 171
GFAP-positive astrocytes	Saline	5853 ± 233	6149 ± 84
MPTP	8257 ± 306⁎	6400 ± 211

Numbers of Iba1-positive microglial cells and GFAP-positive astrocytes in the SNpc of mice were significantly increased 2 days after MPTP, but remained fairly unchanged in mice receiving HMGB1 neutralizing antibody (Anti-HMGB1-AB). Data are mean ± SEM. generated from 4 to 6 mice per group.

⁎ p < 0.05, compared to MPTP-treated mice receiving HMGB1 neutralizing antibody (Newman–Keuls post-hoc test).
